# Predicting health-related quality of life two years post-diagnosis across seven cancer types: using machine learning to identify vulnerable patients

**DOI:** 10.1007/s11136-026-04165-4

**Published:** 2026-02-12

**Authors:** Willemijn F. Oudijk, Belle H. de Rooij, Koen J. van Benthem, Rampal S. Etienne, Simone Oerlemans, Helena M. Verkooijen, Katja K. H. Aben, Geraldine R. Vink, Anne M. May, Floortje Mols, Dimitris Katsimpokis, Nicole P. M. Ezendam

**Affiliations:** 1 Department of Research and Development, Netherlands Comprehensive Cancer Organisation, Utrecht, The Netherlands; 2 CoRPS - Center of Research On Psychological Disorders and Somatic Diseases, Department of Medical and Clinical Psychology, Tilburg University, Tilburg, The Netherlands; 3 Groningen Institute for Evolutionary Life Sciences, University of Groningen, Groningen, The Netherlands; 4 Universitair Medisch Centrum Utrecht, Divisie Beeld, Utrecht, The Netherlands; 5 Science Department IQ Health, Radboud University Medical Centre, Nijmegen, The Netherlands; 6 Department of Medical Oncology, University Medical Center Utrecht, Utrecht University, Utrecht, The Netherlands; 7 Julius Center for Health Sciences and Primary Care, University Medical Center Utrecht, Utrecht University, Utrecht, The Netherlands

**Keywords:** Health-related quality of life (HRQoL), Cancer survivorship, Predictive modelling, Machine learning, Vulnerability factors, Long-term consequences

## Abstract

**Purpose:**

Cancer survivors often experience long-term consequences affecting their Health-Related Quality of Life (HRQoL). Sociodemographic factors, clinical characteristics, and health-related behaviours influence HRQoL, making some individuals vulnerable to adverse HRQoL. This study develops linear regression and machine learning models to predict HRQoL two-year post-diagnosis and to identify key vulnerability factors.

**Methods:**

This longitudinal study included data of survivors of seven cancer types. Nineteen predictor variables were derived from questionnaires completed within three months post-diagnosis (baseline) from the Netherlands Cancer Registry. Linear regression, random forest, XGBoost, neural network, and Support Vector Machine (SVM) regressors were employed to predict the EORTC QLQ-C30 summary score 1.5–2.5 years post-diagnosis. Permutation testing assessed vulnerability factors.

**Results:**

The analyses included 4,538 individuals. All models achieved similar R^2^ (0.3) and RMSE (9) scores. Linear regression, random forest, XGBoost, and SVM models identified lower physical, cognitive, and emotional functioning at diagnosis, along with more comorbidities, cancer type (especially endometrial), and higher BMI as the top vulnerability factors. Treatment, age, and education were not associated with vulnerability. All models tended to overestimate low HRQoL which might be due to the limited number of observations with low HRQoL values.

**Conclusions:**

The predictors used in this analysis explained only 30% of the variation in long-term HRQoL. Similar to previous studies predicting HRQoL in cancer, these predictors miss crucial information. Baseline functioning, comorbidities, cancer type and BMI appeared to be the key vulnerability factors. Future studies should prioritize accurate prediction of low HRQoL scores.

**Supplementary Information:**

The online version contains supplementary material available at 10.1007/s11136-026-04165-4.

## Introduction

Globally, the number of cancer survivors is expected to increase in the upcoming years due to an increase in cancer incidence, and improvements in treatment, diagnostics and the organisation of healthcare [1]. The majority of these survivors are at risk for a number of adverse consequences following a cancer diagnosis and treatment including fatigue, pain, emotional symptoms, appetite loss and dyspnoea [2–4]. These consequences can influence an individual’s well-being and health-related quality of life (HRQoL) [5].

Research has demonstrated that HRQoL is influenced by sociodemographic and clinical factors as well as health-related behaviours, indicating that certain individuals may be more vulnerable to experiencing adverse HRQoL outcomes. For instance, illiteracy, unemployment, being female, being single, lacking social interactions, older age, comorbidities, African American ethnic background, and impaired mental health have been shown to contribute to increased vulnerability for impaired HRQoL among cancer survivors [6–10]. Certain health-related behavioural factors, such as alcohol consumption and smoking were associated with HRQoL in head and neck cancer patients [11]. Patient-reported functioning at diagnosis is linked to overall survival in both young adults with cancer [12] and lung cancer patients undergoing chemotherapy [13]. Because baseline functioning influences overall survival, it might also affect long-term HRQoL. Determining which factors and behaviours influence HRQoL serves a crucial role in identifying individuals who are vulnerable to adverse HRQoL outcomes and who might benefit from interventions.

As the complexity and volume of data on cancer survivors continues to grow, machine learning has emerged as a powerful tool in cancer research. Machine learning is predominantly applied to aid in assessing patient susceptibility for cancer prevention [15], facilitating (early) diagnosis [16], predicting patient prognosis regarding disease recurrence [17] and survival [18] as shown in recent reviews [19–21]. In contrast, applications of ML to predict long-term patient-reported outcomes, such as HRQoL, remain limited. Existing studies have generally focused on a single cancer type and often lack integration of behavioral and psychosocial predictors, which are crucial to understanding HRQoL [22, 23]. This suggests that there is a research gap regarding the application of machine learning to predict the long-term consequences of cancer across various types of cancer.

This study aimed to develop linear regression and machine learning models to predict long-term HRQoL outcomes in cancer survivors. In particular, we aimed to predict the HRQoL at two years after diagnosis for survivors of ovarian, endometrial, colon, rectal, bladder, prostate, and breast cancer, which are among the most prevalent solid tumors with relatively high survival rates, and involve diverse clinical characteristics and treatment modalities. The models will incorporate demographic factors, clinical characteristics, health-related behaviours and functioning at time of diagnosis across various cancer types and compare these with a simple reference model. Additionally, we aimed to identify cancer survivors who are vulnerable to adverse HRQoL outcomes by identifying the most important factors contributing to poor HRQoL. These insights will aid clinicians in personalizing HRQoL prognosis and identifying vulnerable cancer survivors who may benefit from additional early-stage clinical support [24, 25].

## Methods

### Study design

We used data of multiple prospective studies within the Patients Reported Outcomes Following Initial treatment and Long term Evaluation of Survivorship (PROFILES) registry [26]. Clinical and sociodemographic data of the Netherlands Cancer Registry (NCR) were linked to these studies. For the patient-reported outcome (PRO) data collection, patients received information and were invited to participate via a letter by their (former) treating physician [26–31]. We made a selection from the PROFILES registry to obtain colon and rectal cancer survivors from the Prospective Dutch Colorectal Cancer (PLCRC) cohort [27] and the Patient-Reported Outcomes in Colorectal Cancer (PROCORE) cohort [32]; ovarian and endometrial cancer survivors from the Registration system Oncological GYnaecology (ROGY) cohort [28]; bladder cancer survivors from the Insight into Bladder Cancer Care (BlaZIB) cohort [29]; prostate cancer survivors from the Insight into Prostate Cancer Care (ProZIB) cohort [30]; and finally breast cancer survivors from the Utrecht cohort for Multiple BReast cancer intErvention studies and Long-term evaLuAtion (UMBRELLA [31]) (Appendix Table 1). In this study, cancer survivor was defined as an individual being alive after a cancer diagnosis, regardless of whether they were cured of cancer [33].

### Patient selection

However, for the purposes of the study, we selected the individuals who had completed a questionnaire within three months of diagnosis and one other between 1.5 and 2.5 years after diagnosis. So, each included patient completed two questionnaires. As such, our study population consists of individuals who survived at least 1.5 years post-diagnosis. Finally, we excluded individuals that were deceased within 2.5 years after diagnosis and individuals with metastatic cancer at initial diagnosis (stage IV). Our study does not capture long-term outcomes beyond 2.5 years.

### Study measures

#### Predictor variables

Sociodemographic characteristics included highest completed educational level (lower/primary education, secondary education (high school), secondary vocational school, vocational education or university), and partner status (married/living together or single/divorced), which were self-reported via the questionnaires. Age at diagnosis (in years) and sex (male or female) were collected through the NCR.

Health-related behaviours including smoking habits (never, former or current smoker), alcohol consumption (never, former or current drinker) and Body Mass Index (BMI; weight in kg/ height in m^2^) were self-reported via the questionnaires.

Clinical characteristics included cancer stage according to the TNM classification [34], treatment (systemic therapy, surgery and radiotherapy (yes or no); whether the individual was undergoing treatment at the time of completing the baseline questionnaire (yes or no)) and cancer type (colon, rectal, ovarian, endometrial, bladder, prostate, and breast cancer) were collected through the NCR. The number of comorbidities (no-, one- or more than one) were self-reported within three months after diagnosis, using a modified version of the Charlson Comorbidity Index [35].

HRQoL was measured via EORTC QLQ-C30 [36]. This is a 30-item questionnaire that consists of 15 scales: five multi-item functional scales (physical, role, social, emotional, and cognitive functioning), nine multi- and single-item symptom scales (fatigue, pain, nausea or vomiting, dyspnoea, insomnia, appetite loss, constipation, diarrhoea, and financial difficulties), and a two-item global health status/QoL scale [37]. Raw scores for each multi-item scale are linearly transformed, where higher scores represent better functioning or worse symptoms [36]. As this resulted in a finite set of possible values between 0 and 100, the scales were treated as discrete variables (Appendix Table 2). For this study, we included the functional scales reported within three months after diagnosis, where higher scores indicate better functioning.

#### Response variable

The response variable was the EORTC QLQ-C30 summary score reported between 1.5- and 2.5-years post-diagnosis (ranging from 0 to 100, higher score indicates better HRQoL) [36]. Known group comparison analyses showed good validity and responsiveness to change over time has found to be good [38, 39]. The summary score was calculated as the average of the functional and symptom scales, excluding financial difficulties and global health status/QoL scores [40–42]. We considered the summary score as a continuous variable because it can take on more than 1,000 distinct values.

### Data exploration and preprocessing

Initially a backward filling imputation method was employed to estimate BMI, comorbidities, highest educational level, partner status, alcohol consumption, and smoking habits in case of missing values. This means that an individual’s baseline variable was imputed using the first available non-missing variable value from a subsequent questionnaire. The remainder of the missing values needed to be estimated for which we employed the random forest based MissRanger imputation algorithm [43]. This algorithm considers the relationships between all predictor variables (except for the response variable) to predict the missing data. We used Predictive Mean Matching (PMM) in missRanger for imputation to ensure realistic and plausible values. To evaluate the imputation method, analyses were also performed on complete case data, i.e., a subset of data with only observations without any missing values. All data selection and preprocessing steps were done using R 4.2.2 (2022-10-31) [44].

### Model development

Multiple models were developed to predict the QLQ-C30 summary score. First, we built a linear regression model as an easy-to-interpret reference model. Furthermore, we explored tree-based algorithms, including random forest regressor [45] and eXtreme Gradient Boosting regressor. Subsequently, we implemented a Support Vector Machine (SVM) for regression. Finally, we developed a feedforward neural network [46]. Additionally, for each model we included the possibility to synthetically increase the few individuals with low HRQoL values in the training dataset, by oversampling them using the Synthetic Minority Oversampling Technique (SMOTE [47]) for regression [48], the artificial instances are created based on real data points. This study was conducted and reported in accordance with the TRIPOD-AI guidelines [49]. These models were developed using Python 3.11.1 [50] with the sklearn [51], XGBoost [52], and TensorFlow [53] libraries.

How well a model’s prediction matches unseen data is defined as the generalizability of the model [54]. To assess the generalizability and performance of the models, we used shuffle split cross-validation with 100 iterations for all predictions (Appendix Fig. 1). For each iteration, data were randomly divided, with 80% allocated to the training dataset and the remaining 20% to the testing dataset. The training data were used directly to train the random forest and XGBoost regressors. Because the other methods are sensitive to data standardization, with standardization typically improving model performance [55, 56], the training data were standardized beforehand for the linear model, the SVM, and the neural network.

Hyperparameters are external parameters of machine learning methods that need to be set manually. Nested cross-validation using a randomized grid search (Appendix Table 3) was used for this [57]. This involved splitting the total dataset in multiple folds into training (80%) and testing (20%) data. The training dataset was further split into training (80%) and validation (20%) subsets. The inner training subset was used to fit the models under different hyperparameter settings, and the validation subsets were used for their performance. The outer testing data was used for evaluating the final model’s performance. This nested cross-validation ensured that the validation data for tuning was distinct from the testing data. To tune the parameter that dictates the maximum number of training cycles without improvement before halting the training process in the neural network model, we included an additional step. In this step, 20% of the training dataset were allocated as validation data. Then, when the model performance on the validation data stopped improving, the training process was terminated.

### Model evaluation

We evaluated model performance by plotting model predictions against true values and by quantification of prediction errors. For the quantitative analyses we employed Root Mean Squared Error (RMSE) and R-squared (R^2^) on the test dataset. The RMSE measures differences between model predictions and observed (true) values on the same scale as the response variable. When the RMSE is 0, predictions perfectly match observed values, and there is no upper limit to its value. To put the RMSE value in perspective, we also calculate the relative error R^2^, i.e., the variance explained by the model compared to a null model that simply returns the average for each test data point. R^2^ quantifies the proportion of variance in the response variable that can be explained by the model. When it has a value of 1, it means that predictions and true values correlate perfectly, while when it has a value of 0, there is no correlation between the two. In simple linear regression models, on training data, the R^2^ value is identical to the square of the correlation between observed and predicted values. Because the metrics are calculated on test data alone, the model could perform worse than the null model, in which case negative values of R^2^ would emerge. To ensure reliability of our evaluation, all metrics were calculated across all iterations of the cross-validation procedure and then averaged to obtain overall performance.

### Feature importance

Feature importance was examined for the reference linear regression model, the random forest, XGBoost regressors and the SVM model as these models and the most-promising machine learning models using permutation testing [58]. The values of a single feature were shuffled in the test data. The model’s drop in R^2^ after shuffling the feature is an indication of its importance for the predictions. Permutation testing was conducted for each feature in every iteration. The results were then gathered and combined across the 100 iterations without summarizing to preserve iteration-specific variability allowing its analysis. The final linear regression model was trained on the entire dataset and the beta coefficients were used to determine the direction of the topmost important features, as identified through permutation testing. Model development and feature importance was done using Python 3.11.1 [50], including the sklearn library [51].

## Results

### Survivors’ characteristics

Approximately 45% of the initial population was included in the analyses (Appendix Fig. 2). Exclusions were related to the absence of a baseline questionnaire within the specified timeframe (30% of the total), missing data regarding the response variable (23% of the total), or death within 2.5 years since diagnosis. This resulted in a final study population of 4,538 survivors of 7 cancer types.

Survivors were on average 66 years old (9.58 SD), and half (51%) of the individuals had colon or rectal cancer (Table 1; Appendix Fig. 3). Appendix Table 4 contains a full breakdown by tumour type.

**Table 1:** Survivors’ characteristics including the imputed data

Characteristic	Survivors (*n* = 4538)
Age at diagnosis, min; mean ± SD; max	24; 66.17 ± 9.58; 94
Sex, No. (%)	
Female	1510 (33)
Male	3028 (67)
BMI, mean (SD)	26.61 (4.58)
Education, No. (%)	
Lower/primary education	256 (6)
Secondary education (high school)	1408 (31)
Secondary vocational education	1219 (27)
Higher vocational education, university	1655 (36)
Partner, No. (%)	
Married/living together	3762 (83)
Single/divorced	776 (17)
Smoking, No. (%)	
Never	1295 (29)
Previous	2572 (57)
Current	671 (15)
Alcohol consumption, No. (%)	
Never/former drinker	2129 (47)
Current drinker	2409 (53)
Comorbidities, No. (%)	
No comorbidities	1552 (34)
One comorbidity	1372 (30)
More than one comorbidity	1614 (36)
Tumor type, No. (%)	
Ovarian cancer	30 (1)
Endometrial cancer	62 (1)
Colon cancer	1389 (31)
Rectal cancer	930 (20)
Bladder cancer	761 (17)
Prostate cancer	928 (20)
Breast cancer	438 (10)
Stage (TNM), No. (%)	
I	1984 (44)
II	1234 (27)
III	1320 (29)
Under treatment at baseline, No. (%)	1327 (29)
Treatment received, No. yes (%)	
Systemic therapy (chemotherapy, targeted therapy and/or immunotherapy)	558 (12)
Received surgery	3270 (72)
Radiotherapy	1312 (29)
Functional scales at baseline, mean (SD)	
Role functioning	80.43 (27.42)
Emotional functioning	81.89 (18.27)
Cognitive functioning	88.91 (16.47)
Physical functioning	89.35 (15.01)
Social functioning	86.13 (20.46)
QLQ-C30 summary score	
Baseline, median (IQR), min–max	90.21 (80.98–95.73), 19.62–100
Two years post-diagnosis, median (IQR), min–max	92.74 (85.13–97.44), 26.67–100

### Model performance in predicting HRQoL two years post-diagnosis

**Fig. 1:**
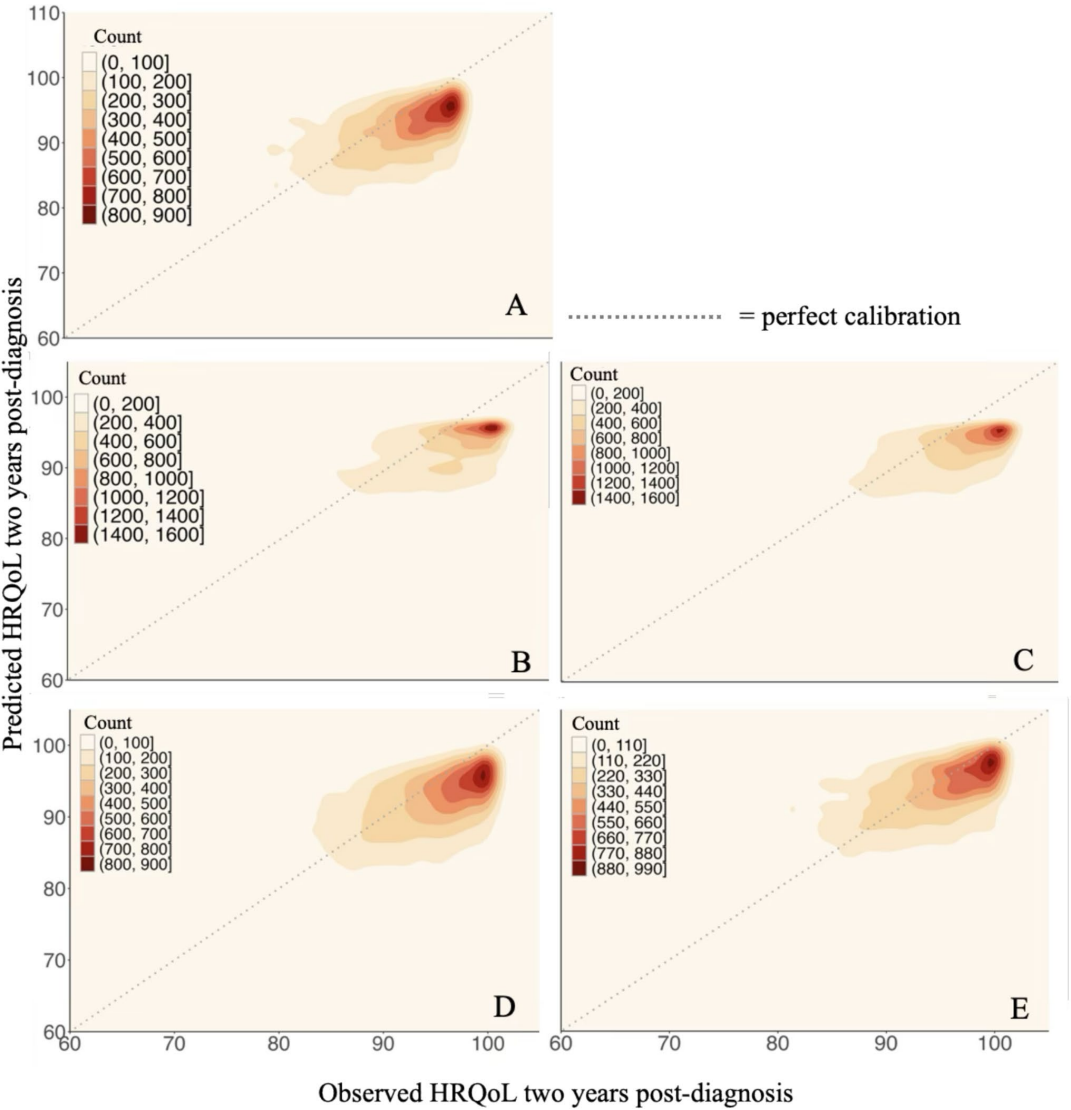
Observed vs. predicted HRQoL values two years post-diagnosis across seven tumour types. Kernel density estimations are shown based on shuffle split cross-validation with 100 iterations. Colours indicate the density of data points across combinations of observed and predicted HRQoL values, with darker shades representing higher concentrations. Each plot corresponds to a different model: **A** Linear regression model, **B** Random forest regressor, **C** XGBoost regressor, **D** Neural network, and **E** SVM

**Fig. 2:**
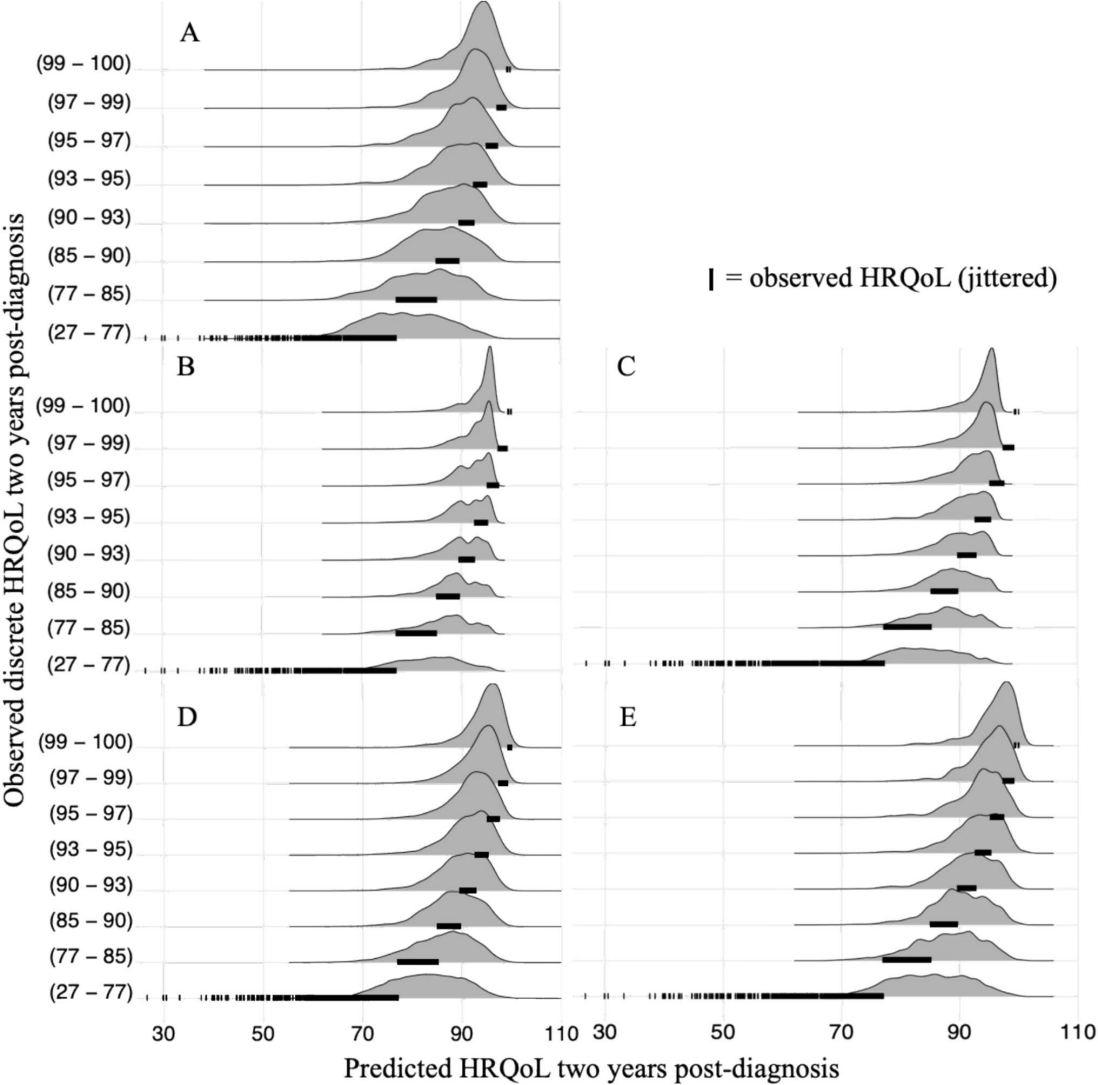
Predicted vs. observed discrete HRQoL values two years post-diagnosis across seven tumour types. Model predictions are shown for each discretised observed HRQoL group based on shuffle split cross-validation with 100 iterations. The discretisation was done using quantile-based grouping to ensure an equal number of observations per group. For each group the observed HRQoL values are represented with jittered points. Each plot corresponds to a different model: **A** Linear regression model, **B** Random forest regressor, **C** XGBoost regressor, **D** Neural network, and **E** SVM

The reported results incorporate hyperparameters tuning (Appendix Table 3). The reference linear regression model achieved a testing RMSE score of 8.9 and a testing R^2^ of 0.3 (Table 2). The XGBoost model performed best in terms of both RMSE and R^2^, but the performance metrics were fairly similar across the various models, with average testing RMSEs ranging from 8.9 and 9.1, and average testing R^2^ values between 0.28 and 0.30. Although there are some minor differences in performances of the models, when the 95% confidence intervals are taken into consideration, that show substantial overlap, these differences are not statistically significant. Overall, hyperparameter tuning mostly fixed overfitting of the random forest and XGBoost regressors. Analyses were conducted using both the imputed dataset and the complete case dataset to assess the validity of the imputation method. The results were highly similar across the two datasets (Table 2 and Appendix Table 5). The subset with complete case data resulted in 3528 individuals, thus 1010 individuals had missing values in their data. The similar performance indicates that imputation preserved the underlying structure in the data. Across all models, most data points occurred at high values for both the observed and the predicted HRQoL (Fig. 1). These high-density regions fell slightly below the perfect calibration line, suggesting that the models tended to underestimate high true values. To investigate this, we visualized predictions for discrete groups of the response variable, where the response variable was discretised into eight quantile-based groups, each containing approximately the same number of data points (Fig. 2). The groups consisted of increasingly smaller ranges of values, indicating a higher concentration of outcome values at high HRQoL levels, thus suggesting that most outcome values were skewed toward the higher end. The group with observed HRQoL values from 27 to 77, were estimated too high by all the models, while the models underestimated the maximum (100) observed values. In the case of the linear regression model (Fig. 2A), the neural network (Fig. 2D) and the SVM model (Fig. 2E) some predictions exceeded the maximum HRQoL score of 100. This is because these models typically do not enforce any specific output constraints and are thus not bounded by a maximum. The synthetically generated individuals with low HRQoL by SMOTE did not improve the models’ performances (Appendix Table 6). The random forest and XGBoost regressors (Fig. 2B and C) exhibited the worst underestimations. Overall, all models tended to predict within a narrow range of values across all eight groups.

**Table 2:** Average and standard deviation of training and testing performance metrics for each model over shuffle split cross-validation with 100 iterations for predicting HRQoL two years post-diagnosis across seven tumour types

Model	Test RMSE	Train RMSE	Test R^2^	Train R^2^
Linear regression	8.968 ± 0.298	8.662 ± 0.074	0.303 ± 0.031	0.345 ± 0.008
Random forest	9.042 ± 0.340	8.074 ± 0.081	0.297 ± 0.029	0.430 ± 0.008
XGBoost	8.897 ± 0.322	8.685 ± 0.077	0.304 ± 0.023	0.344 ± 0.007
Neural network	8.998 ± 0.32	8.393 ± 0.153	0.288 ± 0.029	0.387 ± 0.019
SVM	9.124 ± 0.370	8.950 ± 0.090	0.278 ± 0.028	0.301 ± 0.008

R^2^ values correspond to the fraction of variance in the response variable that is explained by the model. It has a maximum value of 1, indicating perfect correlation between predicted and true values. For simple linear regression R^2^ values for training data correspond to the square of the correlation between predicted and true values, e.g., if in simple regression the correlation between observed and predicted values is 0.55 for the training data, the R^2^ value would be 0.303

### Feature importance

**Fig. 3:**
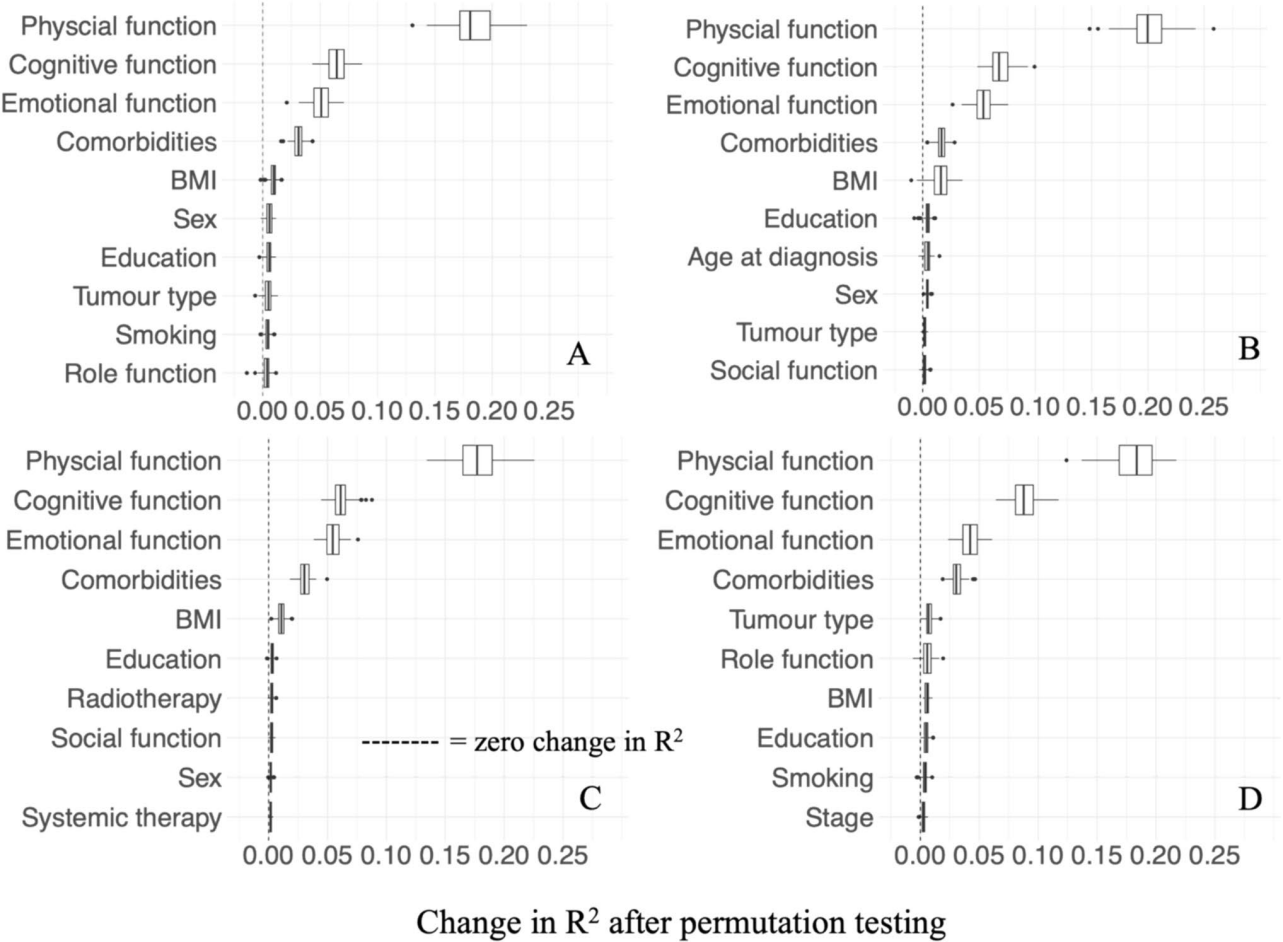
The top 10 most important features over shuffle split cross-validation with 100 iterations for predicting HRQoL two years post-diagnosis across seven tumour types. Feature importance was determined based on permutation testing. Each box plot displays the distribution of changes in R^2^ after permutation testing per feature across 100 iterations of shuffle split cross validation. The central line inside each box represents the median. The boxes represent the interquartile range with the boxes’ edges representing the 25th and 75th percentile of the data. The whiskers extend to the minimum and maximum values within a range of 1.5 times the interquartile range. The black points beyond this range are outliers. Each plot corresponds to a different model: **A** Linear regression model, **B** Random forest regressor, **C** XGBoost regressor, **D** SVM regressor

**Fig. 4:**
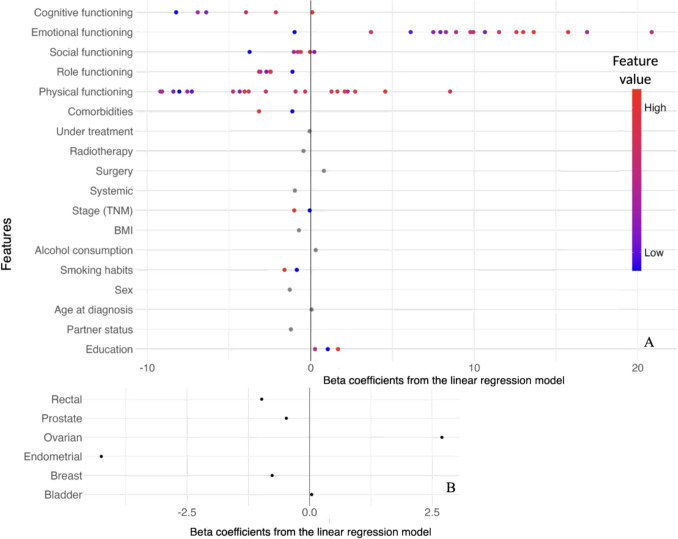
Beta coefficients from the linear regression model for each feature used to predict HRQoL two years post-diagnosis across seven tumour types. The coefficients are calculated relative to a reference category for the categorical predictors. The zero line reflects this reference category. The reference category includes the lowest available functioning scores, absence of comorbidities, not receiving treatment at baseline, no radiotherapy, surgery, or systemic therapy, stage 1 cancer, never or former alcohol drinker, never smoked, male sex, married or living together with a partner, and lower/primary education level. Panel **A** presents the beta coefficients for all available predictors; color indicates the feature value, where red shows higher values of the predictors and blue lower (e.g., cognitive functioning has seven discrete levels, each depicted as a dot (relative to the reference level, i.e., the lowest functioning score). For example, the first blue dot for the feature cognitive functioningindicates that a low value of cognitive functioning (as indicated by the blue color) is associated with a negative beta coefficient (location on the x-axis), and thus a negative effect on HRQoL two year post-diagnosis). Panel **B** focuses on the beta coefficients for each tumour type with colon cancer as reference category.

The top four features were consistent across the reference linear regression model (Figs. 3A, 4A) and the machine learning models (Fig. 3B, D), namely lower physical functioning, emotional functioning, cognitive functioning, and more comorbidities at the time of diagnosis predict poorer HRQoL. For the reference linear regression model (Figs. 3A, 4A), higher BMI predicts poorer HRQoL as the fifth most important feature, which was also true for both tree-based models (Fig. 3B and C), the fifth most important feature for the SVM model was cancer type (Fig. 3D). Endometrial cancer exhibited the strongest negative association with HRQoL compared with colon cancer (Fig. 4B). For the remaining features, permutation testing resulted in a change in R^2^ of close to zero (overview in Appendix Table 7).

## Discussion

This prospective study demonstrated that only about 30% of the variance in HRQoL two years post-diagnosis could be predicted for individuals with any of seven different cancer types, based on their characteristics at diagnosis. Notably, the machine learning models did not outperform the simpler linear regression model. Across all models, key vulnerability factors consistently included lower physical, cognitive and emotional functioning, as well as more comorbidities. However, all models tended to overestimate the low observed HRQoL values, indicating their current limitations for use in clinical practice.

Our performance metric results showed that the models performed very similar to each other with minor differences in metrics. Within the RMSE scores, the difference between the models’ performances was no more than 0.2 and within the R^2^ scores no more than 0.03.

The similar results between the analysis on the imputed dataset and the complete case data suggests that the imputation method did not alter the relationships within the data or introduce bias, this implies that the imputation methods were effective. Given the minimal impact of the imputation on the results, using the complete case dataset could be preferred to preserve the original dataset. However, by using the imputed dataset more data is retained.

The reference linear regression model achieved similar performance scores to the machine learning models. This suggests that this simpler model might be preferred because it is easier to interpret. Several studies find similar results, where the more recently developed machine learning algorithms performed similarly as the classical regression methods [59–62]. However, other studies have reported that machine learning algorithms can outperform the classical regression methods under certain conditions [63–65]. The limited explanatory power of the predictor variables included in this study may have restricted all models' ability to accurately predict HRQoL. Future research should revisit the selection of predictor variables, incorporating variables such as cancer recurrence, illiteracy [8], unemployment [8], ethnic background [10], and coping mechanisms [66], which a linear model might capture more accurately.

Overall, the models achieved an R^2^ score of around 0.3 which implies that 70% of the variability in HRQoL was left unexplained. While this R^2^ may seem low in comparison to studies predicting clinical outcomes such as survival [67], similar R^2^ values of 0.33 and 0.39 were found in previous studies predicting HRQoL outcomes in cancer patients receiving chemotherapy [68] and in young adult survivors [69], respectively. The low R^2^ in our analysis and previous studies may indicate there is inherent unpredictability in HRQoL outcomes.

An average RMSE of 9 indicates that approximately 95% of the residuals fall between − 18 and + 18. This implies that 95% of the predictions deviate no more than 18 units from the actual HRQoL values. This poor performance may be caused by the relatively few survivors with low HRQoL scores—16% had a score lower than 80. Because of this, the RMSE might be misleading: even if the models predicted high HRQoL values accurately but poorly predicted the few low values, the overall RMSE will still appear relatively small. The large number of near-accurate high-value predictions overshadow the poor low-value predictions. This is especially problematic because the low-value predictions are clinically relevant, as these indicate the vulnerable survivors. Furthermore, survivorship bias, which occurs when only patients who are alive at the study endpoint are included, may have further skewed our results by excluding those with more severe outcomes. While imputation of missing response data could reduce this bias, it could also introduce bias by distorting the true relationships between variables, as it may artificially smooth over the variability in HRQoL outcomes.

The linear regression model, the random forest, XGBoost, and SVM regressors agreed on the topmost important vulnerability factors. Our findings are in line with previous research showing that lower baseline physical functioning, emotional functioning, comorbidities and BMI are related to poor long-term HRQoL outcomes in cancer survivors according to review [71] and prediction [72–75] studies. These findings may simply support the ‘better in is better out’ principle or the cancer and treatment may have more impact on their HRQoL. We did not find studies related to cognitive functioning and HRQoL outcomes. In contrast to other studies, we did not identify other factors to be predictive for HRQoL, such as treatment, age, smoking status, and educational level. It is likely that these factors are closely related to other factors in our models and do not appear to be of additional relevance. Additionally, endometrial and ovarian cancer had the strongest negative impact on long-term HRQoL compared to colon cancer. However, cancer type showed minimal influence on HRQoL in our analysis, suggesting that pooling different cancer types together does not compromise the model's generalizability across tumour types. Furthermore, as shown in previous literature, we saw that baseline functioning (being part of HRQoL), is above all other factors the most important predictor of future HRQoL [23]. Including these variables is clinically relevant, as they reflect early vulnerability and are already available in routine care, supporting timely identification of patients at risk. This implies that it may be relevant for future studies to address changes in HRQoL rather than HRQoL at a single follow-up point. Alternatively, future studies could explore HRQoL one year after diagnosis. This could potentially help in identifying critical periods when patients may need additional support. Lastly, it is crucial to mention that the feature importance present here cannot be given a causal interpretation as the models do not estimate marginal probabilities (e.g., of medical interventions across the whole sample) but only conditional ones (e.g., what is the HRQoL given a person is 89 years old) [76].

Given the complexity of predicting HRQoL, several limitations inherent to this study should be considered. First of all, the proportion of patients with low HRQoL was limited. To tackle this, we synthetically increased the number of individuals with low HRQoL value. This did not improve the models’ performance metrics. This could indicate that these synthetic cases did not accurately capture the true underlying patterns in the data. We recommend that future research efforts focus on improving the recruitment of patients with low HRQoL to participate in prospective studies. A second limitation was that HRQoL had a maximum value constraint, causing the models to underestimate the high observed HRQoL values. To address this, we transformed the response variable using a logit transformation, but overall model performance worsened. Further, although cancer type had a small overall contribution to the explained variance, the large proportion of colorectal cancer patients in our dataset may have skewed our results, potentially limiting the generalizability to other cancer types.

Future research may explore extended generalized linear models as they allow response variables with non-normal error distributions [77]. One possibility for improving both linear regression and machine learning models is through the exploration of different loss functions. Given the observed inaccuracies of the models predicting edge cases, a loss function that specifically penalizes errors occurring at the edges of the response variable could potentially improve the models’ accuracy. Such as the quantile loss function, specifically designed to handle predictions at different quantiles of the response variable’s distribution [78].

Predictive models may be promising in aiding healthcare providers identifying patients with poor prognosis of long-term HRQoL among cancer survivors. Increasingly, survivors complete PROMs in daily clinical care. These can, in combination with other vulnerability factors, be used to predict poor outcomes (e.g., treatment choice, HRQoL, survival) and inform personalized supportive care [79, 80].

In conclusion, this study demonstrated that HRQoL two years post-diagnosis can be predicted with an overall explained variance of about 30%. Similar to previous studies predicting HRQoL in cancer, the predictors included did not accurately capture long-term HRQoL. This indicates that predictors commonly used in prediction studies lack crucial information on long-term HRQoL [81]. Moreover, machine learning models were not better than a simple linear regression model. All models performed poorly in predicting low HRQoL and thus predicting who is vulnerable. Patients with low baseline HRQoL (i.e., physical functioning, cognitive functioning, emotional functioning), more comorbidities, endometrial or ovarian cancer and higher BMI may be more vulnerable for lower HRQoL outcomes and may be in need for early supportive care. Future studies may focus on improving the accuracy of predicting lower HRQoL scores by improving the recruitment of patients with poor HRQoL and by including a broader range of parameters.

## Supplementary Information

Below is the link to the electronic supplementary material.

Supplementary file1 (DOCX 443 KB)

## Data Availability

Data are available through the cohort studies’ corresponding author with the permission of the respective study principal investigator. Restrictions apply to the availability of these data, which were used under license for this study. The R code to generate the figures in this report and to perform data selection, and Python code for all model development is available in: https://github.com/WFOudijk/QoLPredictionWithML.
