# Supplementary material for: Predicting health-related quality of life two years post-diagnosis across seven cancer types: using machine learning to identify vulnerable patients
Source: Qual Life Res. 2026 Feb 12;35(3):67. doi: 10.1007/s11136-026-04165-4 (PMC12901195; doi:10.1007/s11136-026-04165-4)
Supplement: Supplementary file 1 — Supplementary file1 (DOCX 443 KB) [file 11136_2026_4165_MOESM1_ESM.docx]

**Predicting health-related quality of life two years post-diagnosis across seven cancer types: using machine learning to identify vulnerable patients**

**Quality of Life Research**

Willemijn F. Oudijk^1,3^, Belle H. de Rooij^1,2^, Koen J. van Benthem^3^, Rampal S. Etienne^3^, Simone Oerlemans^1^, Helena M. Verkooijen^4^, Katja K.H. Aben^1,5^, Geraldine R. Vink^1,6^, Anne M. May^7^, Floortje Mols^1,2^, Dimitris Katsimpokis^1^, Nicole P.M. Ezendam^1,2^

^1^Department of Research & Development, Netherlands Comprehensive Cancer Organisation, Utrecht, The Netherlands.

^2^CoRPS - Center of Research on Psychological disorders and Somatic diseases, Department of Medical and Clinical Psychology, Tilburg University, Tilburg, The Netherlands

^3^Groningen Institute for Evolutionary Life Sciences, University of Groningen, Groningen, The Netherlands

^4^Universitair Medisch Centrum Utrecht, Divisie Beeld, Utrecht, The Netherlands

^5^Science department IQ Health, Radboud university medical centre, Nijmegen, The Netherlands

^6^Department of Medical Oncology, University Medical Center Utrecht, Utrecht University, Utrecht, The Netherlands

^7^Julius Center for Health Sciences and Primary Care, University Medical Center Utrecht, Utrecht University, Utrecht, The Netherlands.

Corresponding author:

NE: [N.Ezendam@iknl.nl](mailto:N.Ezendam@iknl.nl)

**Appendix Table 1** Overview of the inclusion criteria for the included cohort studies

| **Cohort** | **Cancer type** | **Inclusion years** | **Inclusion criteria** | **Time since diagnosis**  **(months)** | | | | | | | **Time since initial treatment (months)** | | | |
| --- | --- | --- | --- | --- | --- | --- | --- | --- | --- | --- | --- | --- | --- | --- |
|  |  |  |  | 0 | 3 | 6 | 12 | 18 | 24 | 36 | 0 | 6 | 12 | 24 |
| PROCORE | Colorectal | December 2015 - March 2019 | Stage I-III | **X*** |  |  | X |  | **X*** |  |  |  |  |  |
| ROGY | Endometrial / Ovarian | April 2011 - March 2014 | Stage I-III |  |  |  |  |  |  |  | **X*** | X | X | **X*** |
| BlaZIB | Bladder | November 2017 - October 2019 | Stage I-III | **X*** |  | X | X |  | **X*** |  |  |  |  |  |
| ProZIB | Prostate | October 2015 - March 2016 | Stage I-III | **X*** |  |  | X |  | **X*** |  |  |  |  |  |
| PLCRC | Colorectal | June 2013 - January 2023 | Stage I-III | **X*** | X | X | X | X | **X*** |  |  |  |  |  |
| Umbrella | Breast | January 2014 - May 2023 | Stage I-III | **X*** | X | X | X | X | **X*** |  |  |  |  |  |

**Appendix Table 2** Possible values for the categorical functioning scales from the EORTC QLQ-C30 questionnaire. These multi-item functioning scales were linearly transformed. This resulted in a finite set of values and were thus treated as discrete variables.

| Functioning scale | Value |
| --- | --- |
| Role functioning | 0.0, 16.7, 33.3, 50, 66.7, 83.8, 100 |
| Emotional functioning | 0.0, 8.3, 16.7, 22.2, 25.0, 33.3, 41.7, 44.4 50.0, 55.6, 58.3, 66.7, 75.0, 77.8, 83.3, 88.9, 91.7, 100.0 |
| Cognitive functioning | 0.0, 16.7, 33.3, 50, 66.7, 83.8, 100 |
| Physical functioning | 6.7, 13.3, 20.0, 26.7, 33.3, 40.0, 46.7, 50.0, 53.3, 60.0, 66.7, 73.3, 75.0, 77.8, 80.0, 83.3, 86.7, 88.9, 91.7, 93.3, 100.0 |
| Social functioning | 0.0, 16.7, 33.3, 50, 66.7, 83.8, 100 |


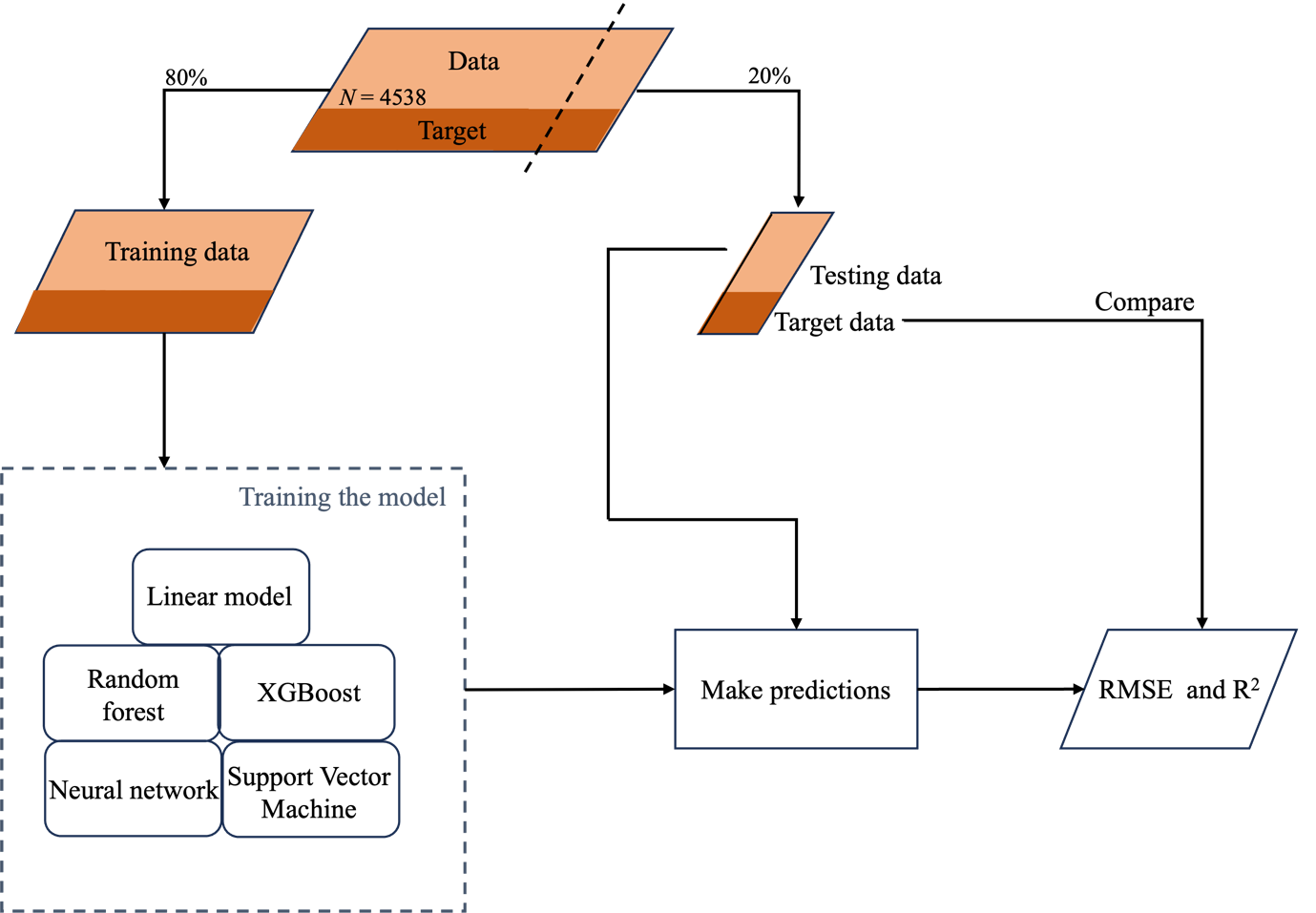


**Appendix Fig. 1** Study methodology pipeline of a single iteration within the 100-iteration shuffle split cross validation framework. In each iteration, the data were randomly divided into training data (80%) and testing data (20%). The target data represents the outcome variable

**Appendix Table 3** Overview of the hyperparameters tuned for the various models

| Model | Hyperparameter | Range | Final value |
| --- | --- | --- | --- |
| Random forest | Number of trees in the forest (*n_estimators*) | 100, 311, 522, 733, 944, 1155, 1366, 1577, 1788, 2000 | 100^a^ |
|  | Number of features to consider (*max_features*) | None, sqrt, log2 | Sqrt^a^ |
|  | Maximum depth of the tree (*max_depth*) | None, 1 – 15 | 6 |
|  | Minimum number of samples required to split an internal node (*min_samples_split*) | 13 - 20 | 18 |
|  | Minimum number of samples required to be a leaf node (*min_samples_leaf*) | 1, 2, 4 | 1^a^ |
|  | Bootstrapping when building trees (*bootstrap*) | True, False | True^a^ |
| XGBoost | Number of trees (*n_estimators*) | 1, 3, 5, 7, 9, 11, 13, 15, 17, 20 | None^a^ |
|  | Step size at which the optimizer makes updates to the weights (*learning_rate*) | 0.01, 0.02, 0.03, 0.04, 0.05, 0.06, 0.07, 0.08, 0.09, 0.1 | 0.05 |
|  | Maximum depth of a tree (*max_depth*) | None, 1 - 5 | 2 |
|  | Minimum loss reduction required for a split (*gamma*) | 0, 71, 142, 214, 285, 357, 428, 500, 571, 642, 714, 785, 857, 928, 1000 | 0^a^ |
|  | L2 regularization term on weights (*reg_lambda*) | 0, 71, 142, 214, 285, 357, 428, 500, 571, 642, 714, 785, 857, 928, 1000 | 1^a^ |
|  | L1 regularization term on weights (*reg_alpha*) | 0, 71, 142, 214, 285, 357, 428, 500, 571, 642, 714, 785, 857, 928, 1000 | 0^a^ |
| SVM | Regularization parameter (*C*) | 0.1, 0.31, 0.52, 0.73, 0.94, 1.16, 1.47, 1.58, 1.79, 2.0 | 1.47 |
|  | Tolerance for stopping criterion (*tol*) | 0.001, 0.112, 0.223, 0.334, 0.445, 0.556, 0.667, 0.778, 0.889, 1.0 | 0.001^a^ |
|  | Degree of the polynomial kernel function (*degree*) | 1 - 10 | 3^a^ |
|  | Specifies the kernel type (*kernel*) | Linear, poly, sigmoid, rbf | Linear |
|  | Defines the error margin (*Epsilon*) | 0.001, 0.112, 0.223, 0.334, 0.445, 0.556, 0.667, 0.778, 0.889, 1.0 | 0.1^a^ |
| Neural network | Optimizer algorithm | Adam, SGP | Adam |
|  | Optimizer learning rate (*learning_rate* in Adam function) | 0.00001, 0.0001, 0.001, 0.01 | 0.00001 |
|  | Number of layers | 1 – 10 | 6 |
|  | Number of neurons per layer | Less no. neurons per layer: 10, 20, 30 | 10 (first layer: 110, last: 70) |
|  | Number of epochs with no improvement after which training will be stopped (*patience)* | 1 - 8, 10, 20, 30, 40, 50 | 40 |
|  | Minimum change to qualify as improvement (*min_delta*) | 0.001, 0.01, 0.05, 0.1, 0.3, 0.4, 0.5, 0.6 | 0.001 |

^a^ *Default value*


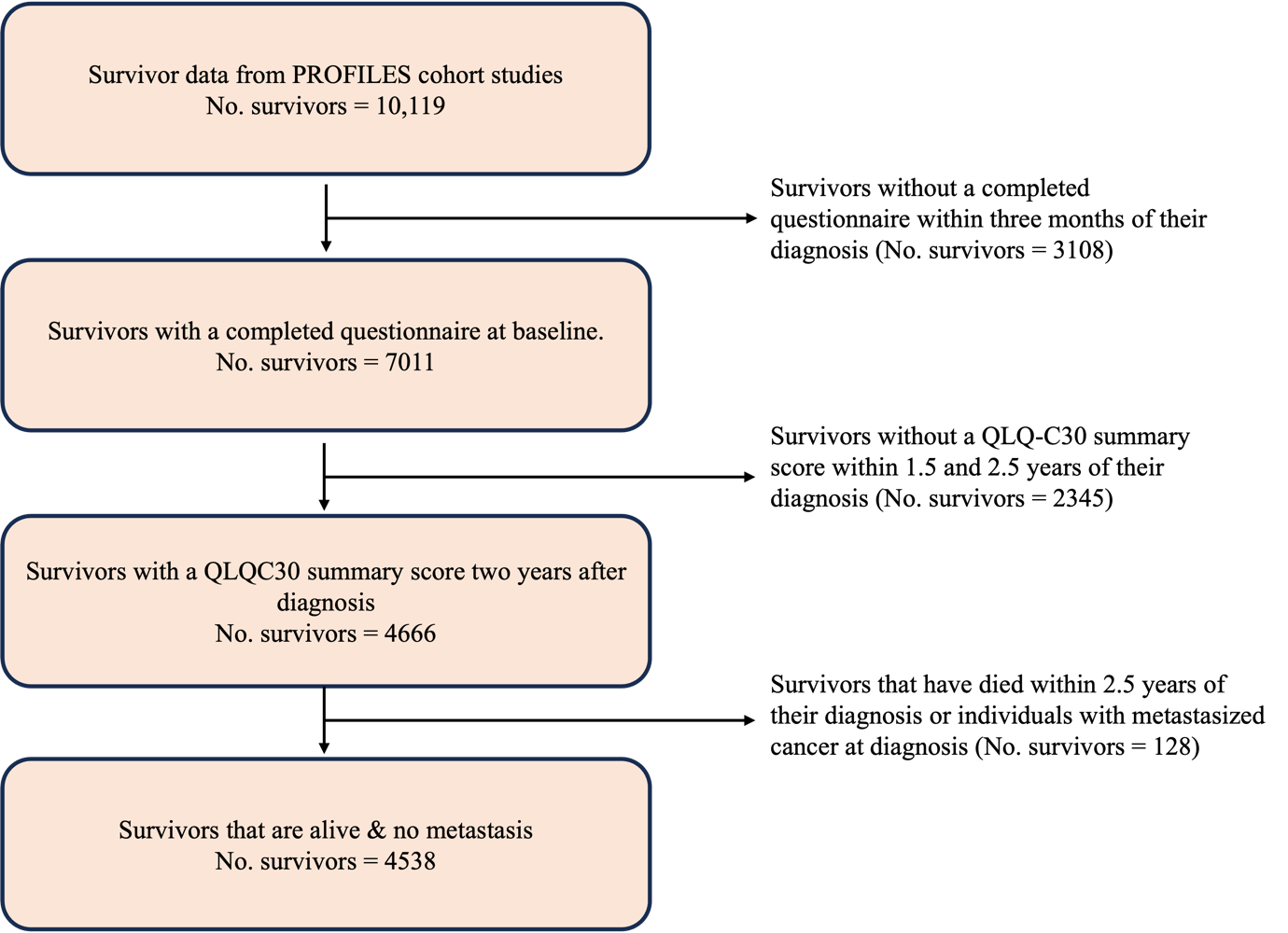


**Appendix Fig. 2** Study population selection procedure

**
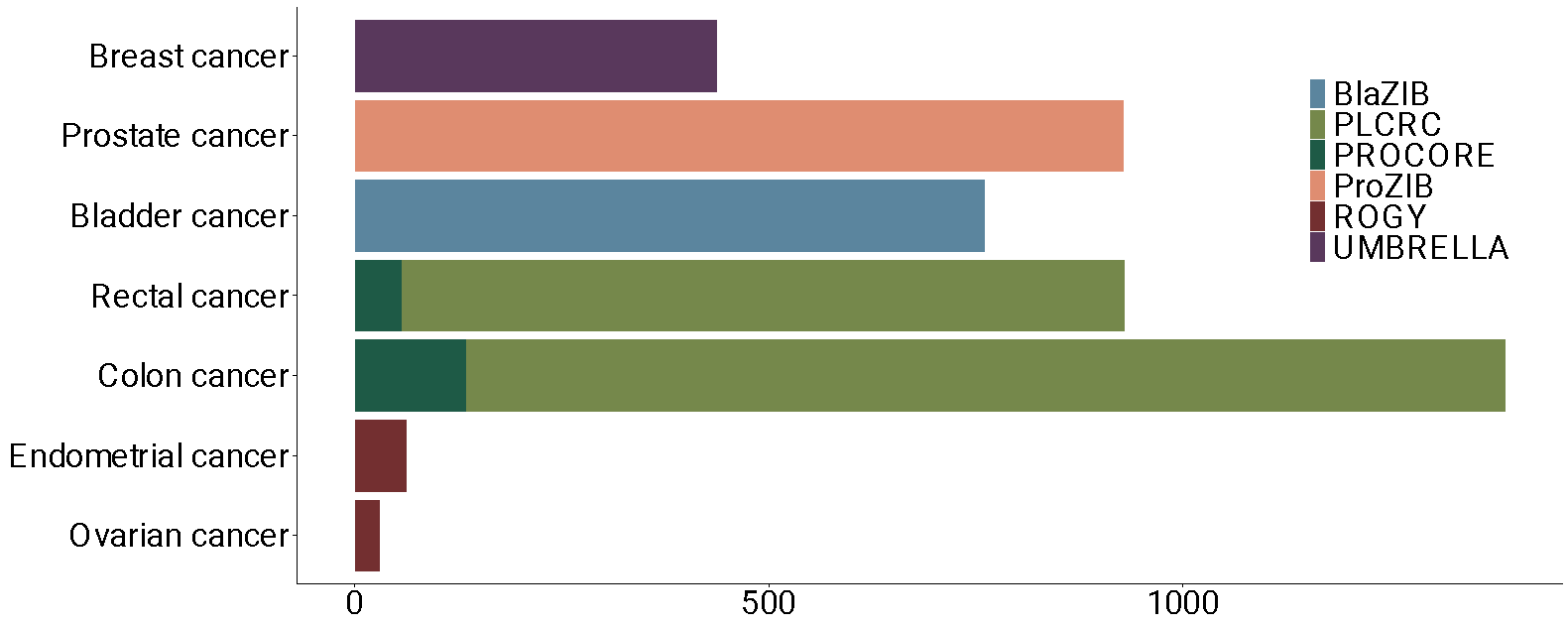
Appendix Fig. 3** Tumour types and cohort distribution

**Appendix Table 4** Survivors’ characteristics including the imputed data by tumour type. Note: percentages are rounded, which sometimes result in totals not adding up to exactly 100%

| Characteristic | Survivors (n = 4538) | Ovarian | Endometrial | Colon | Rectal | Bladder | Prostate | Breast |
| --- | --- | --- | --- | --- | --- | --- | --- | --- |
| Age at diagnosis, min; mean ± SD; max | 24; 66.17 ± 9.58; 94 | 45; 60.37 ± 8.87; 77 | 41; 66.27 ± 8.50; 88 | 24; 66.20 ± 9.62; 93 | 29; 64.45 ± 9.36; 91 | 35; 70.57 ± 8.90; 94 | 45; 68.67 ± 6.76; 87 | 30; 57.20 ± 9.27; 78 |
| Sex, No. (%) |  |  |  |  |  |  |  |  |
| Female | 1510 (33) | 30 (100) | 62 (100) | 576 (41) | 268 (29) | 138 (18) | 0 (0) | 2 (0.5) |
| Male | 3028 (67) | 0 (0) | 0 (0) | 813 (59) | 662 (71) | 623 (82) | 928 (100) | 436 (99.5) |
| BMI, mean (SD) | 26.61 (4.58) | 25.10 (5.14) | 29.89 (5.67) | 26.58 (5.08) | 26.32 (4.19) | 26.41 (3.60) | 27.01 (4.45) | 26.12 (4.74) |
| Education, No. (%) |  |  |  |  |  |  |  |  |
| Lower/primary education | 256 (6) | 4 (13) | 4 (6) | 87 (6) | 46 (5) | 64 (8) | 46 (5) | 5 (1) |
| Secondary education (high school) | 1408 (31) | 22 (73) | 51 (82) | 444 (32) | 291 (31) | 181 (24) | 307 (33) | 112 (26) |
| Secondary vocational education | 1219 (27) | 4 (13) | 7 (11) | 333 (24) | 221 (24) | 319 (42) | 245 (26) | 90 (21) |
| Higher vocational education, university | 1655 (36) | 0 (0) | 0 (0) | 525 (38) | 372 (40) | 197 (26) | 330 (36) | 231 (53) |
| Partner, No. (%) |  |  |  |  |  |  |  |  |
| Married/living together | 3762 (83) | 24 (80) | 52 (84) | 1113 (80) | 784 (84) | 629 (83) | 811 (87) | 349 (80) |
| Single/divorced | 776 (17) | 6 (20) | 10 (16) | 276 (20) | 146 (16) | 132 (17) | 117 (13) | 89 (20) |
| Smoking, No. (%) |  |  |  |  |  |  |  |  |
| Never | 1295 (29) | 17 (57) | 31 (50) | 498 (36) | 251 (27) | 120 (16) | 350 (38) | 28 (6) |
| Previous | 2572 (57) | 11 (37) | 23 (37) | 807 (58) | 529 (57) | 526 (69) | 493 (53) | 183 (42) |
| Current | 671 (15) | 2 (7) | 8 (13) | 84 (6) | 150 (16) | 115 (15) | 85 (9) | 227 (52) |
| Alcohol consumption, No. (%) |  |  |  |  |  |  |  |  |
| Never/former drinker | 2129 (47) | 12 (40) | 32 (52) | 880 (63) | 661 (71) | 138 (18) | 257 (28) | 149 (34) |
| Current drinker | 2409 (53) | 18 (60) | 30 (48) | 509 (37) | 269 (29) | 623 (82) | 671 (72) | 289 (66) |
| Comorbidities, No. (%) |  |  |  |  |  |  |  |  |
| No comorbidities | 1552 (34) | 14 (47) | 23 (37) | 407 (29) | 434 (47) | 188 (25) | 239 (26) | 247 (56) |
| One comorbidity | 1372 (30) | 9 (30) | 22 (35) | 432 (31) | 274 (29) | 185 (24) | 364 (39) | 86 (20) |
| More than one comorbidity | 1614 (36) | 7 (23) | 17 (27) | 550 (40) | 222 (24) | 388 (51) | 325 (35) | 105 (24) |
| Tumor type, No. (%) | 4,538 | 30 (1) | 62 (1) | 1389 (31) | 930 (20) | 761 (17) | 928 (20) | 438 (10) |
| Stage (TNM), No. (%) |  |  |  |  |  |  |  |  |
| I | 1984 (44) | 11 (37) | 58 (94) | 413 (30) | 237 (25) | 526 (69) | 487 (52) | 252 (57) |
| II | 1234 (27) | 5 (17) | 0 (0) | 455 (32) | 177 (19) | 184 (24) | 247 (27) | 166 (38) |
| III | 1320 (29) | 14 (47) | 4 (6) | 521 (38) | 516 (55) | 51 (7) | 194 (21) | 20 (5) |
| Under treatment at baseline, No. (%) | 1327 (29) | 21 (70) | 16 (26) | 305 (22) | 299 (32) | 451 (59) | 99 (11) | 136 (31) |
| Treatment received, No. yes (%) |  |  |  |  |  |  |  |  |
| Systemic therapy (chemotherapy,  targeted therapy and/or immunotherapy) | 558 (12) | 23 (77) | 1 (2) | 48 (3) | 285 (31) | 45 (6) | 1 (0.1) | 155 (35) |
| Received surgery | 3270 (72) | 30 (100) | 62 (100) | 1389 (100) | 841 (90) | 173 (23) | 337 (36) | 438 (100) |
| Radiotherapy | 1312 (29) | 0 (0) | 20 (32) | 3 (0.2) | 476 (51) | 77 (10) | 318 (34) | 418 (95) |
| Functional scales at baseline, mean (SD) |  |  |  |  |  |  |  |  |
| Role functioning | 80.43 (27.42) | 55.55 (29.14) | 81.19 (18.22) | 76.50 (30.68) | 81.13 (28.00) | 79.22 (25.86) | 90.16 (19.40) | 74.51 (27.42) |
| Emotional functioning | 81.89 (18.27) | 77.49 (25.36) | 80.33 (18.82) | 81.11 (18.79) | 80.36 (18.68) | 83.43 (17.30) | 85.29 (16.47) | 78.27 (19.10) |
| Cognitive functioning | 88.91 (16.47) | 78.33 (25.20) | 85.75 (17.29) | 88.09 (17.52) | 90.32 (15.20) | 89.81 (15.45) | 90.89 (14.34) | 83.90 (19.14) |
| Physical functioning | 89.35 (15.01) | 69.78 (22.47) | 89.33 (7.09) | 88.81 (15.60) | 91.41 (14.02) | 84.79 (17.20) | 92.09 (12.47) | 90.15 (13.26) |
| Social functioning | 86.13 (20.46) | 72.78 (24.16) | 79.3 (24.08) | 83.50 (22.60) | 84.98 (20.75) | 86.09 (19.45) | 93.41 (14.85) | 83.45 (20.00) |
| QLQ-C30 summary score |  |  |  |  |  |  |  |  |
| Two years post-diagnosis, mean (SD) | 89.49 (10.72) | 85.50 (13.44) | 84.29 (13.23) | 89.86 (10.81) | 89.48 (10.67) | 88.61 (10.85) | 91.09 (9.64) | 87.54 (11.18) |

**Appendix Table 5** Average and standard deviation of training and testing performance metrics on complete case data (i.e., a subset of data with only observations without any missing values) for each model over shuffle split cross-validation with 100 iterations for predicting HRQoL two years post-diagnosis across seven tumour types.

| Model | Test RMSE | Train RMSE | Test R^2^ | Train R^2^ |
| --- | --- | --- | --- | --- |
| Linear regression | 9.140 ± 0.417 | 8.749 ± 0.104 | 0.289 ± 0.040 | 0.340 ± 0.010 |
| Random forest | 9.083 ± 0.406 | 8.074 ± 0.096 | 0.284 ± 0.028 | 0.441 ± 0.007 |
| XGBoost | 9.111 ± 0.407 | 8.732 ± 0.101 | 0.294 ± 0.032 | 0.343 ± 0.009 |
| Neural network | 9.532 ± 0.566 | 9.146 ± 0.169 | 0.226 ± 0.076 | 0.279 ± 0.022 |
| SVM | 9.352 ± 0.401 | 9.063 ± 0.105 | 0.260 ± 0.031 | 0.291 ± 0.010 |

**Appendix Table 6** Average and standard deviation of training and testing performance metrics on data with synthetically generated individuals with low HRQoL using SMOTE for each model over shuffle split cross-validation with 100 iterations for predicting HRQoL two years post-diagnosis across seven tumour types.

| Model | Test RMSE | Train RMSE | Test R^2^ | Train R^2^ |
| --- | --- | --- | --- | --- |
| Linear regression | 9.198 ± 0.309 | 9.407 ± 0.114 | 0.260 ± 0.040 | 0.374 ± 0.009 |
| Random forest | 9.202 ± 0.289 | 8.724 ± 0.090 | 0.263 ± 0.029 | 0.463 ± 0.008 |
| XGBoost | 9.154 ± 0.318 | 9.265 ± 0.110 | 0.268 ± 0.031 | 0.393 ± 0.008 |
| Neural network | 9.327 ± 0.427 | 10.588 ± 0.773 | 0.236 ± 0.060 | 0.288 ± 0.098 |
| SVM | 9.035 ± 0.349 | 9.680 ± 0.122 | 0.284 ± 0.036 | 0.338 ± 0.010 |

**Appendix Table 7** The full list of features according to their importance for the linear regression model, the random forest, XGBoost, and SVM regressors over shuffle split cross validation with 100 iterations for predicting HRQoL two years post-diagnosis across seven tumour types. Feature importance was determined based on permutation testing

| Model | Linear regression | Random forest | XGBoost regressor | SVM |
| --- | --- | --- | --- | --- |
| Feature 1 | Physical Functioning | Physical Functioning | Physical Functioning | Physical Functioning |
| Feature 2 | Cognitive Functioning | Cognitive Functioning | Cognitive Functioning | Cognitive Functioning |
| Feature 3 | Emotional Functioning | Emotional Functioning | Emotional Functioning | Emotional Functioning |
| Feature 4 | Comorbidities | Comorbidities | Comorbidities | Comorbidities |
| Feature 5 | BMI | BMI | BMI | Tumour type |
| Feature 6 | Sex | Education | Education | Role Functioning |
| Feature 7 | Education | Age at diagnosis | Radiotherapy | BMI |
| Feature 8 | Tumour type | Sex | Social Functioning | Education |
| Feature 9 | Smoking habits | Tumour type | Sex | Smoking habits |
| Feature 10 | Role Functioning | Social Functioning | Systemic therapy | Stage |
| Feature 11 | Partner status | Systemic therapy | Partner status | Social Functioning |
| Feature 12 | Stage | Smoking habits | Role Functioning | Alcohol consumption |
| Feature 13 | Surgery | Radiotherapy | Age at diagnosis | Partner status |
| Feature 14 | Systemic therapy | Partner status | Smoking habits | Radiotherapy |
| Feature 15 | Social functioning | Role Functioning | Tumour type | Sex |
| Feature 16 | Radiotherapy | Surgery | Stage | Surgery |
| Feature 17 | Alcohol consumption | Under treatment | Surgery | Systemic therapy |
| Feature 18 | Age at diagnosis | Stage | Under treatment | Under treatment |
| Feature 19 | Under treatment | Alcohol consumption | Alcohol consumption | Age at diagnosis |
